# Perspectives for the future in China: challenges and opportunities facing animal teaching, research, and industry

**DOI:** 10.1093/af/vfaa019

**Published:** 2020-07-23

**Authors:** Jingdong Yin, Zhengpeng Zhu

**Affiliations:** 1 College of Animal Science and Technology, China Agricultural University, Beijing, China; 2 Department of Animal Nutrition and Feed Science, Sichuan Tequ Agriculture and Animal Husbandry Technology Group Co., Ltd, Sichuan, China

**Keywords:** animal science research, China, developmental status, livestock industries

**Implications**

- Chinese animal industries face challenges in finding graduates with the appropriate training for the needs of a dynamic industry.
- The agricultural universities of China are working on methods to strengthen the specialized abilities of students to meet industry needs.
- The Chinese government has increased funding to encourage faculty and students to devote energy to animal science research not only in tradition field, but also in some new cross–disciplinary areas, for example, artificial intelligence applications.
- These challenges have accelerated the formation of mutually beneficial relationships between the industries and universities but there is a long way to go.

## The Current Situation

Following more than 20 years of rapid development, China is the largest producer of meat and eggs and the third-largest producer of milk in the world. Since 2016, the animal industry in China has reached a plateau in which the yields of meat, eggs, and milk increased by no more than 3% per year (Department of International Cooperation and Agricultural Trade Promotion Center, Ministry of Agriculture and Rural Affairs of People’s Republic of China, 2018). Meanwhile, in contrast to pursuing a large annual output of animal products, the Chinese government has increasingly emphasized the establishment of rules and regulations, as well as standards to push the industry toward sustainable, efficient, and environmentally friendly production of safe, high-quality animal products. According to a personal survey of the enterprises, producers are required to spend about 10–14 million RMB (approximately 1.4–2.0 million US dollars), which is equivalent to 20% of the total investment on infrastructure and facilities of a pig farm with a size of 500 breeding sows to comply with environmental protection regulations. This capital requirement also poses a high financial threshold for new investors.

Chinese (mainland) imports of livestock and poultry products reached 36.2 billion U.S. dollars in 2019 (National Bureau of Statistics of China, 2019). The Sino-U.S. trade agreement, signed on January 15, 2020, will result in an increased volume of imported livestock and poultry products (more than 10 billion U.S. dollars of meat, milk, and egg products) appearing on the Chinese domestic market with a competitive edge on price (Office of the U.S. Trade Representative, 2020). This added volume will substantially increase the competitive pressure on the Chinese livestock industries.

More importantly, animal disease epidemics heavily depress the development of a sustainable animal industry in China. For example, the spread of African swine fever (ASF) in China since 2018 caused producers to lose tens of billions (U.S. dollar) losses during 2019. In addition, hundreds of pig farms, especially mid- and small-sized farms, went out of business.

## The Opportunities for Education

To strengthen the ability of agricultural industries to withstand risk, more companies are beginning to integrate the feed industry, animal husbandry, and processing. Only powerful and integrated companies, along with some unique mid- and small- size enterprises, can survive and attain profit in China. A huge and integrated animal industry needs hundreds of thousands of employees involved in the primary and supporting industries. However, many young people prefer to move to urban areas to enjoy a convenient lifestyle rather than work on remote and isolated farms, causing a deficiency in farm labor throughout China. Considering the huge demands for qualified employees with well-trained professional knowledge, skills and interdisciplinary background, employers are willing to offer high salaries to qualified employees. Unfortunately, the candidates with a traditional background in animal science regardless of degree are at a relative disadvantage when applying for these high-salary positions due to the absence of cross-disciplinary training.

To strengthen the graduates’ abilities to deal with challenges and cooperate with colleagues from across branches in integrated enterprises, designing the animal science curriculum and supporting courses for the future has become a primary issue. Establishment of new courses reflecting the transformation of the industry to develop students with the following abilities would be welcomed.

1. Application of artificial intelligence in the animal industries;
2. Understanding experimental design, statistical analyses, interpretation of data, and scientific communication skills (oral and written);
3. Training in biotechnology, including cell and molecular biology, genetic engineering, and fermentation engineering;
4. Expertise and skill in nutritional strategies to keep animals healthy, minimize agricultural environmental impact and emissions, and promote production efficiency;
5. Skills in control and prevention of infectious diseases;
6. Animal product quality control and monitoring;
7. Design of animal husbandry buildings and auxiliary infrastructure, including design of facilities, feed processing and conveyor systems, environmental control systems, and other engineering skills;
8. Excellent interdisciplinary background covering animal husbandry, veterinary science, feed processing, environmental monitoring, financial accounting and analysis, and so forth.

## The Opportunities for Research

The Chinese government also annually identifies scientific issues on the frontier, including the common problems impacting the development of animal industries. They also create funding for projects addressing these priority issues through the National Natural Science Fund or other projects of national scientific programs.

Issues concerning the development of animal genetic resources and breeding, control and prevention of epidemics, feed production/processing/efficient utilization, animal immunity, environmental stress and animal health, animal nutrition, application of artificial intelligent in the animal industry, animal waste processing and environmental protection, and animal behavior and animal welfare attract more financial support than has previously been available from the government and from the industries.

According to the Statistical Bulletin on National Investment in Science and Technology for 2018 (National Bureau of Statistics of China, 2019), the total expenditure of research and development in China reached 1,967.8 billion RMB (approximately 281 billion U.S. dollars), which is an 11.8% increase. Expenditures on basic research were more than 109 billion RMB (approximately 15.6 billion U.S. dollars). The ratio of research and development expenditure to gross domestic product increased by 2.19%, bringing the average level of Organization for Economic Cooperation and Development (OECD) close to 2.37% but yet far below than that of 2.79% in the United States. However, as one of three main funding recipients, Chinese universities received 7.4% of total research and development expenditures, ranked after industries (more than 77%) and state-run institutions (13%).

**Figure 1.**
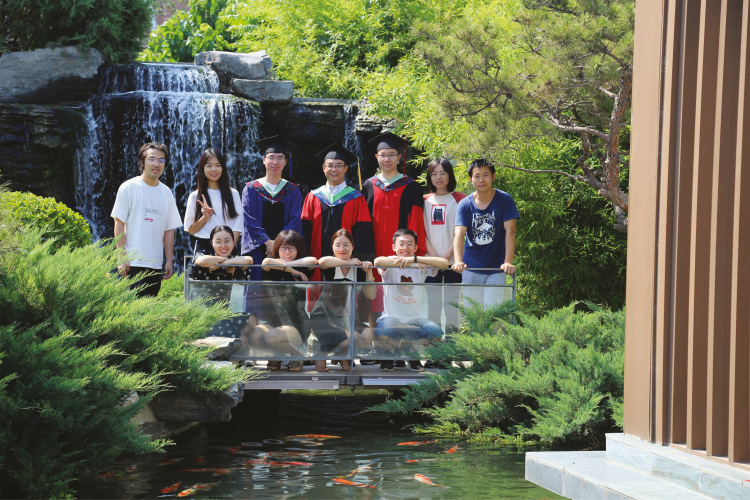
Celebrating the future available to well-trained Chinese animal sciences graduates.

The government understands that investment in high-level scientific research activities of animal science and development of the full-fledged potential and ability/skills of students who will have careers in animal husbandry and related works is money well spent. Additionally, university administrators must do their best to provide facilities that encourage faculty and students to upgrade student skill sets for professional careers of the future because employment opportunities exist for well-trained graduates (Figure 1). To facilitate effective research in the field of animal/veterinary science, adequate project funding, maintenance of infrastructure, including animal research farms and well-equipped laboratories, and well-trained technicians are also needed.

## Conclusions

In summary, it is of great importance for the university education system in China to cultivate students with professional knowledge and know-how to serve the rapidly transforming animal industry. This must be done by increasing the needed funds to promote multidisciplinary training and high-quality scientific research activities.

## About the Author

Dr Jingdong Yin is a professor in the College of Animal Science and Technology, China Agricultural University (CAU). He was a postdoctoral fellow at CAU, where he studied animal nutrition and muscle biology. Dr Yin earned his PhD in animal nutrition and feed science from Chinese Academy of Agricultural Sciences in 2000. From 2012 to 2013, he studied stem cell development and regeneration at the University of California at Los Angeles as a visiting scholar. His current research focuses on the mechanisms that mediate myogenesis/adipogenesis homeostasis, skeletal muscle insulin sensitivity, skeletal muscle development (including myofiber composition and intramuscular fat deposition), and the relationship between nutritional metabolism in muscle and meat quality in livestock.

Dr Zhengpeng Zhu is Vice President of Sichuan Tequ Agriculture and Animal Husbandry Technology Group Co., Ltd. He joined Cargill Animal Nutrition, Beijing, China, as Technology Application Manager (Swine) and Noah Pork Segment Marketing Manager. He also worked at New Hope Liuhe Co., Ltd, Chengdu Center, China, as the technology department GM and Feed Product Department GM. Zhu earned his PhD in animal nutrition and feed science from China Agricultural University and the University of Missouri (Columbia). Dr Zhu’s research interests focus on feed ingredient evaluation and swine nutrition.
